# Detection of Heart Rate through a Wall Using UWB Impulse Radar

**DOI:** 10.1155/2018/4832605

**Published:** 2018-04-01

**Authors:** Hui-Sup Cho, Young-Jin Park

**Affiliations:** Intelligent Devices and Systems Research Group, DGIST, Daegu, Republic of Korea

## Abstract

Measuring the physiological functions of the human body in a noncontact manner through walls is useful for healthcare, security, and surveillance. And radar technology can be used for this purpose. In this paper, a new method for detecting the human heartbeat using ultra wideband (UWB) impulse radar, which has advantages of low power consumption and harmlessness to human body, is proposed. The heart rate is extracted by processing the radar signal in the time domain and then using a principal component analysis of the time series data to indicate the phase variations that are caused by heartbeats. The experimental results show that a highly accurate detection of heart rate is possible with this method.

## 1. Introduction

The demand for noninvasive and contactless measurement of physiological functions is constantly on the rise, and such technology is useful for monitoring the conditions of hospitalized patients [1] as well as detecting individuals for security, surveillance, and military purposes. Research has been carried out to utilize the radar technology, which is mainly used in aeronautical navigation and military applications, to acquire physiological information from the human body [2, 3]. Ultra wideband (UWB) impulse radar has low exposure risk for the human body as well as low power consumption and high compatibility with peripheral equipment [4]. Thus, UWB impulse radar has been considered as a useful tool for measuring biological information. The signal processing method for extracting biometric information from the radar signal reflected from the human body is dependent on the radar pulse shape. This method can be regarded as a process of searching for critical factors that can describe the characteristics of the physiological functions effectively. Moreover, the radar signal is processed for extracting the physiological information in various domains such as time domain and frequency domain [5, 6]. There are previous works on detecting the heartbeats of a human body through a wall with UWB impulse radar [7–9], but these studies deal with radar signals in the frequency domain, so these methods can only express the dominant frequency component during a particular time interval and cannot provide time domain characteristics. In this paper, to overcome these limitations, a new method that can both detect human heartbeats and extract their transient characteristics through walls by utilizing a UWB impulse radar is proposed. In order to accomplish this, the radar signals are accumulated at specific time intervals, and the set of accumulated signals is sent through a bandpass filter designed to remove the components reflected by the wall. The heartbeat information is extracted through a principal component analysis (PCA) of the filtered data set. An experiment for verifying the performance of the proposed method is also carried out, and it is confirmed that the heart rate can be accurately extracted with this method.

## 2. UWB Impulse Radar Signal

**Figure 1:**
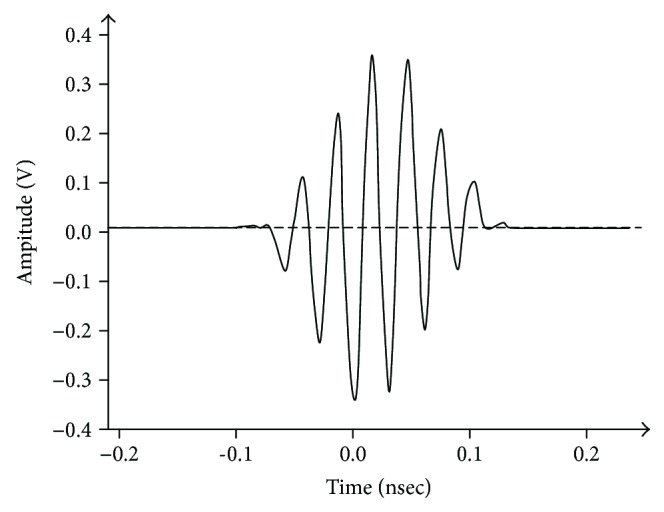
The shape of a radar output pulse.

**Figure 2:**
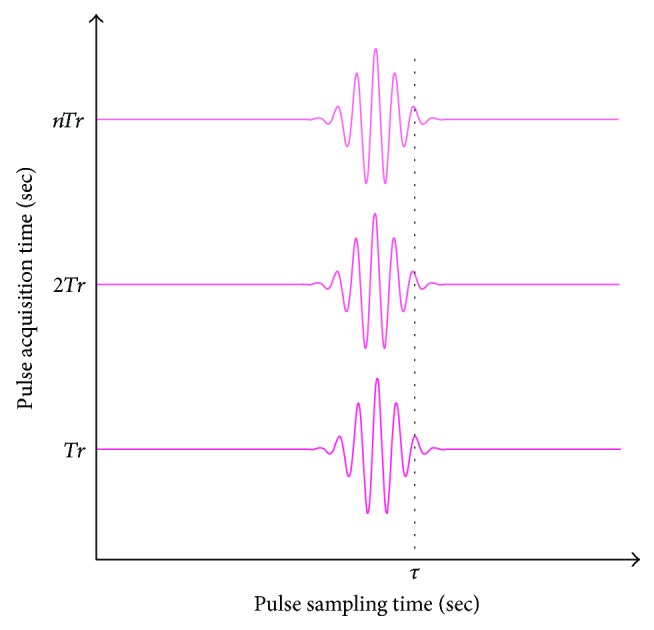
Conceptual shape of radar pulses used in mathematical expression.

A radar transceiver, NVA6201 [10], which emits pulses via an antenna and digitizes the pulses coming back from the target using a strobed sampling method, is employed in the experiment. Each pulse is in the form of a sine wave with a Gaussian envelope and a width of less than 0.4 ns, and the pulse has a center frequency of 6.8 GHz and a bandwidth of 2.3 GHz. The shape of an output pulse is shown in Figure 1. A clay brick wall is used in the present experiment, and the pulses reflected from the body return to the radar antenna through the wall. As the pulse reaching the human body loses energy due to the wall, the amplitude of the component acquired by the transceiver is reduced. The attenuation of the signal corresponding to the frequency differs according to the wall material [11]. Pulses that pass through the body's surface interact with tissues. Most of the energy of the pulses that travel into body tissues is absorbed by the tissues, and the components that are reflected at the interface of each tissue and released into the air are negligible. Therefore, it can be assumed that the pulses arriving at the antenna are mainly reflected from the interfaces of either the wall or the body surface. In the present study, the radar pulse reflected by the wall is assumed as a stationary signal with time-invariant characteristics, and the component reflected from the human body is a nonstationary signal that has phase variations caused by the respiration and heartbeats. Hence, considering these properties, it is necessary to separate these two components. The conceptual images used to describe the shapes of the radar pulses captured at the receiving antenna mathematically are shown in Figure 2. The range sampled at the sampling rate of the receiver that samples the pulse is referred to as the pulse sampling time, and the cross-range sampled at the pulse repetition frequency, which is the frequency at which the pulse is emitted, is referred to as the pulse acquisition time in this paper. According to Ren et al. [12], the radar pulse emitted by the antenna is expressed as

(1) $$\begin{array}{c} x_{tx}\left(t,{mT}_{tx}\right)=g\left(t,{mT}_{tx}\right)\sin\left(2\pi f_{c}t\right), \end{array}$$

where g(*t*) and *f*_c_ denote the Gaussian function and the center frequency of the sine wave, respectively, and *t* denotes the time variable in pulse sampling time. *T*_*tx*_ represents the period for emitting pulses to the target, and *mT*_*tx*_ represents the time at which the *m*th pulse is emitted. In addition, the expression for the pulses received by the radar system after being reflected from the human body can be found in [12] and is

(2) $$\begin{array}{c} x_{rx}\left(t,{nT}_{rx}\right)=g\left(t-T_{d},{nT}_{rx}\right)\sin\left(2\pi f_{c}\left(t-T_{d}\right)\right), \end{array}$$

(3) $$\begin{array}{c} T_{d}=2\frac{\left[R+A_{r}\sin\left(2\pi f_{r}{nT}_{rx}\right)+A_{h}\sin\left(2\pi f_{h}{nT}_{rx}\right)\;\right]}{c}, \end{array}$$

where *T*_*rx*_ represents the period for collecting pulses reflected from the target, and *nT*_*rx*_ represents the time at which the *n*th pulse is collected. In addition, *T*_d_ represents the time delay that occurs on the path along which the pulse is reflected off the target and back to the radar system and includes *A*_r_, *A*_h_, *f*_r_, *f*_h_, and *c* that represent the respiration amplitude, heartbeat amplitude, respiration frequency, heartbeat frequency, and speed of light, respectively. Assuming that the antenna is in close contact with body surface and the subject holds breathing in order to make the equation simpler, *A*_r_ = 0 and *R* = 0. Under this condition, as shown in Figure 2, at the moment when *t* is a specific time *τ*, (2) is expressed as

(4) $$\begin{array}{c} x_{rx}\left(\tau ,{nT}_{rx}\right)=g\left(\tau -T_{d},{nT}_{rx}\right)\sin\left(2\pi f_{c}\left(\tau -T_{d}\right)\right), \end{array}$$

(5) $$\begin{array}{c} T_{d}=2\frac{\left[A_{h}\sin\left(2\pi f_{h}{nT}_{rx}\right)\;\right]}{c}. \end{array}$$

It can be seen that the g(*τ* − *T*_d_, *nT*_*rx*_) is independent of *nT*_*rx*_, so *T*_d_ determines the shape of the received radar pulse as a function of *nT*_*rx*_ in (4). As a result, the phase changes of the radar signals due to the heartbeats can be observed along the pulse acquisition time axis in the accumulated received signals.

## 3. Previous Work

A study was carried out to extract the heartbeat frequency accurately as well as to observe the change of the heartbeat patterns by processing the radar signals in the time domain [5]. A method using SFCW (stepped frequency continuous wave) radar for approximating the frequencies of the heart rates and the position of persons behind a wall was proposed by Shirodkar et al. [7], but they made no mention of the method's quantitative accuracy. Chia et al. [8] introduced a UWB radar prototype that meets FCC emission limits and can measure the heartbeat and breathing rate of persons behind a wall, but they did not indicate how accurately the prototype could measure heart and breathing rates. Singh et al. [9] applied the short-term Fourier transform (STFT) and singular value decomposition (SVD) methods to radar signals reflected from gypsum walls, wooden doors, and persons behind walls. However, heartbeats could not be discriminated.

## 4. Methodology

### 4.1. Setup for Radar Signal Measurement

**Figure 3:**
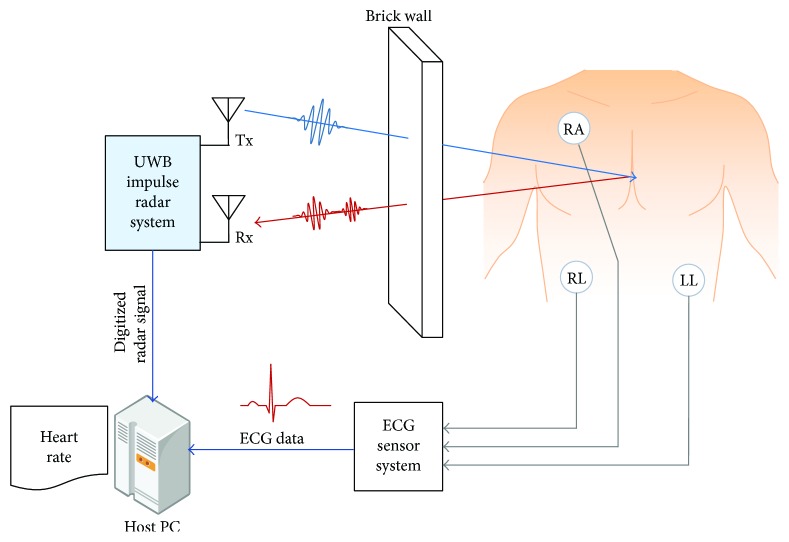
Experimental setup.

The setup for measuring radar signals is shown in Figure 3. Pulses were emitted from the transmitter antenna (Tx), and the pulses coming back from the target were received by the receiver antenna (Rx). The radar pulse emitted from the transceiver has an average power of −12.6 dBm and the antenna amplifies this by 6 dBi. The UWB impulse radar system converted the received radar signals into digital data and transmitted them to the host PC that executed the proposed algorithm. The ECG sensor system was synchronized with the UWB impulse radar system using the same time base, and the ECG heartbeats were compared with those extracted by the radar system as reference points. The power of the received signal is inversely proportional to the fourth power of distance in the radar equation. If the distance to the target is more than 1 m, then signal amplification is needed in the radar pulse transmitter/receiver stage in order to detect the heartbeats. In the present study, it was not appropriate to use signal amplification, because this might have led to excessive human exposure to electromagnetic waves. Therefore, the experimental radar data was measured from human bodies located no further than 1 m from the radar device.

### 4.2. Preprocessing of the Radar Signal

**Figure 4:**
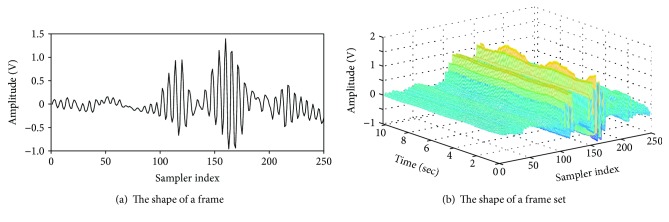
Received radar pulses.

A received pulse was a superposition of all the reflected waves that exhibited changes in both amplitude and phase at a particular moment. Furthermore, considering the movement of the ribcage by breathing and heartbeat, the shape of the pulse became more complicated. The accumulation, using specific time intervals, of the reflected received signals has a phase component. This phase is a function of the time delay that occurs on the path through which the pulse is reflected from the target and back to the radar system. Because both respiration and heartbeat change the time delay, an observation of the accumulated signals along the pulse acquisition time axis reveals phase changes due to these factors as well. The received pulse was output as digital data after being sampled in the transceiver. A set of outputs of each sampler representing the amplitudes of the received pulse is called a “frame.” For detecting the heartbeat, a “frame set,” which was obtained by accumulating 512 frames, was used. The frameset can be viewed as a two-dimensional array consisting of 256 sampler indices and 512 time units. The shape of a frame and a frame set are shown in Figure 4. As mentioned earlier, the time series of each sampler of the frame set contains the phase changes of the radar pulses caused by both the respiration and the heartbeat. The peaks with constant amplitudes near the 120th sampler index in Figure 4(b) are the components reflected by the wall, and some of the ripples near the 170th index in Figure 4(b) are the components reflected by the human body. Specifically, the small and large ripples were due to phase changes caused by heartbeat and breathing, respectively.

### 4.3. Heartbeat Extraction Method

The algorithm described below was used in order to extract heartbeat information in the time domain.

#### 4.3.1. Bandpass Filtering

**Figure 5:**
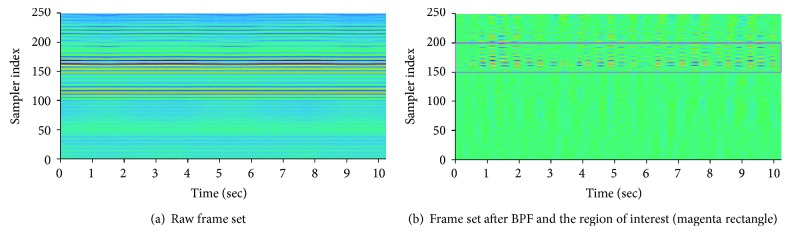
Shape of the frame set before and after BPF (top view).

As the frequency range of a heartbeat is between 1 and 3 Hz [13], the time series of each sampler was passed through a bandpass filter (BPF) that selectively passes the frequency components between 1 and 3 Hz. With this filter, high-frequency noise and low-frequency components originating from stationary targets, such as walls or human respiration, were effectively removed. The function of the bandpass filter can be observed clearly by comparing the frame sets before and after passing through the filter. To accomplish this, two frame sets are shown together in a top view in Figures 5(a) and 5(b), respectively. The peaks observed in the time series of the samplers between the 100th and 125th indices are caused by the peaks of the pulses reflected from the wall, and the peaks observed in the time series of the samplers between the 150th and 180th indices are caused by the peaks of the pulses reflected from the human body. It is clear in Figure 5(b) that the component reflected from the wall was effectively removed near the 120th sampler. It is also apparent that a certain pattern appears in the region between the 150th and 200th samplers, which was a combination of a pattern of heartbeats in the time axis direction and the peak of a frame whose amplitude had been changed by the BPF in the sampler axis direction. To implement the bandpass filter, a fourth order Butterworth filter with cutoff frequencies of 1 Hz and 3 Hz was designed.

#### 4.3.2. Extracting Principal Pattern via Dimensional Reduction of Time Series

**Figure 6:**
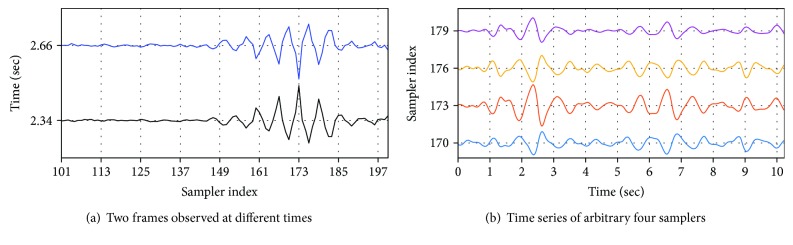
Shape of the frames and the time series after BPF.

The frames in the frame set at 2.34 and at 2.66 s that were passed through the bandpass filter, shown in Figure 5(b), are extracted and shown in Figure 6(a), and the two frames seem to be mirror images of one another. In fact, the frame at 2.34 s corresponds to a case in which the chest cavity was swollen by a heartbeat, and the frame at 2.66 s corresponds to a case in which the chest cavity was reduced, so the phases of the frames are extremely different. It is clear that the phase of the pulse changes as the propagation path of the radar pulse changes. In addition, the phase change of the radar pulse appears in the time series data of each sampler. To visualize this, the time series data of four samplers are shown in Figure 6(b). Since these four samplers correspond to the positive and negative peaks in the frame shown in Figure 6(a), each waveform synchronized with the actual heartbeat of each sampler is inverted vertically. Because there are waveforms synchronized with the actual heartbeats in the time series of the samplers in the region of interest, designated in Figure 5(b) as a magenta rectangle, if a pattern that can represent the multiple time series is found, it is regarded as the heartbeat waveform. In other words, heartbeat waveforms can be found by reducing the dimensions of the time series data. The following procedure is performed for this purpose.

- Construct a matrix with the time series data in the region of interest and call it the “original data set.” From a different perspective, each matrix frame corresponds to an observation of a particular event, and each matrix sampler corresponds to a variable.
- Create a covariance matrix of the original data set.
- Perform eigenvalue decomposition on the covariance matrix in order to find eigenvectors.
- Select the eigenvector with the highest eigenvalue and name it the “principal component.”
- Convert the original data set into one-dimensional data by projecting it onto the principal component vector.

**Figure 7:**
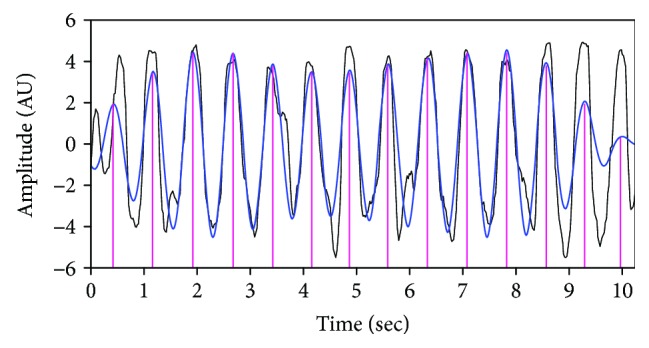
Heartbeats restored from the PCA.

This process is known as PCA [14], and in this paper, the heartbeat waveform is found from the data restored by the PCA. Specifically, the PCA for the time series of 50 samplers (i.e., the 50 × 512 matrix represented by the magenta rectangle in Figure 5(b)) was performed. The shape of the restored signal revealing the phase change due to heartbeat is shown by the black line in Figure 7. It is preferable to detect the heartbeat using patterns found with the PCA of multiple time series data rather than using only one specific time series in the region of interest. Although the time series in the region of interest has a larger signal amplitude than the other samplers do and the waveform synchronized to the heartbeat is evident, each individual time series is susceptible to both quantization errors occurring in the transceiver and noise caused by movement of the subject. Therefore, it is reasonable to use PCA, which can overcome the vulnerabilities of individual time series by considering multiple time series.

#### 4.3.3. Calculating the Heartbeat Intervals

As part of the process of extracting the heartbeat components by removing high-frequency glitches from the restored signal obtained by PCA, the signal was passed through a low-pass filter with an adaptive cutoff frequency, which is a value obtained by adding 0.3 Hz to the dominant frequency of the restored signal. The dominant frequency is obtained by the fast Fourier transform (FFT) of the restored signal. The shape of the signal passing through the LPF is shown by the blue line in Figure 7. Because this signal was synchronized with the heartbeat, the interval of each peak corresponded to the interval of the heartbeat. The temporal position of the extracted heartbeat is indicated by the dotted magenta line. The mean value of every peak interval was estimated as the heart rate.

### 4.4. ECG Data Processing

It is necessary to quantify how accurately the heartbeats detected by the UWB impulse radar follow the actual heartbeats. Therefore, the time at which the R peak occurred in the ECG was compared with the temporal position of the heartbeats extracted by the radar. The accuracy of the proposed method was quantified by calculating the error between these values. For this purpose, the UWB radar system and the ECG sensor system were synchronized at the same time base. The QRS complexes that consisted of successive Q, R, and S waves and were the most obvious parts of the ECG were extracted utilizing the Pan and Tompkins algorithm [15]. The time at which the R peak occurred and the time interval between the R peaks were then used as the reference values. Because a detailed description of the QRS detection algorithm was beyond the scope of this paper, it has been omitted.

## 5. Experiment and Discussion

The setup for the experiment was the one shown in Figure 3, where the clay brick wall was located 50 cm from the antenna of the radar system, and the subject was located 15 cm away from the wall. The subject was breathing normally without sudden movements while sitting in a chair, and electrodes were attached to his or her right arm (RA), left leg (LL), and right leg (RL) to obtain a LEAD II ECG. After emitting radar pulses to each subject, the signals reflected from the body were collected, and the heart rate was extracted using the proposed method for the radar signals. Five subjects with different physical conditions participated in the experiment. During the collection of the radar signal, the ECG was measured and used as a reference point for determining the accuracy of the estimation method. The error rate of beats per minute (BPM) is used as an index of the accuracy of the proposed method. The BPM is obtained by multiplying the heartbeat frequency by 60. The error rate was expressed as

(6) $$\begin{array}{c} Error\;rate=\frac{\left|{BPM}_{Estimate}-{BPM}_{Ref}\right|}{{BPM}_{Ref}}\times 100\left(\%\right), \end{array}$$

where BPM_Estimate_ and BPM_Ref_ represented the BPM extracted from the radar signals and the BPM from the ECG, respectively.

Sixteen frame sets were taken from each subject in the experiment. The average error rate for 80 frame sets was approximately 1.05%. Despite the fact that the heartbeat frequency of each subject had different values, the heart rate could be detected accurately with the proposed method. When a person participates in the experiment as a target, the area of the body surface, where the radar pulses are reflected from, is considered as a factor affecting the power of the received radar signals [5]. However, the results from the experiment showed that a difference in the area had an insignificant effect on the power of the received radar pulse. Therefore, the physical factor can be considered as not affecting the accuracy of the proposed method, which is due to the fact that the subjects are relatively close to the antenna and the opening angles of the antenna are narrow.

**Figure 8:**
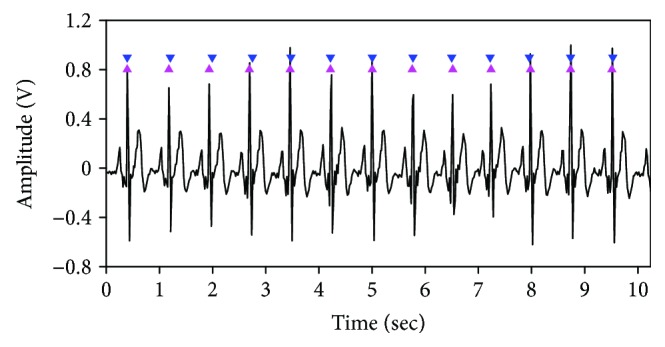
Comparison of heartbeat positions indicated by ECG (magenta upward-pointing triangles) and the positions estimated by the proposed algorithm (blue downward-pointing triangles).

Because comparable studies utilizing UWB impulse radar to measure human heart rate through a wall did not quantify the accuracy of their heart rates, it is difficult to compare the results obtained in this paper directly with those of the other studies. However, some studies using UWB impulse radar to extract a heartbeat without wall penetration [16, 17] found a minimum error rate of 4.0–5.4%, and even in comparison to these studies, the experimental result of this study demonstrated higher accuracy. The researchers of all comparable studies extracted heart rate in the frequency domain. Frequency domain processing carries the limitation that it cannot ensure high frequency and time resolution simultaneously. In contrast, the proposed method has the ability to satisfy two requirements: to provide sufficiently high-frequency resolution and to observe heartbeat changes in the time domain. This was confirmed by placing the positions of the heartbeats detected by the proposed method at the same time intervals as those of the measured ECG. The ECG and the detected heartbeats, where the first beat position was aligned with the first QRS peak of the ECG, are shown in Figure 8. There appear to be slight errors in the third and fourth positions, but the extracted heartbeats follow the actual ECG readings closely.

Heart rate was extracted with high accuracy in experiments with walls such as a clay brick wall, a wooden wall, and a concrete wall with uniform internal structure. However, since the power of the received radar signal was significantly attenuated due to reflection and scattering of the radar pulse in a wall composed of materials with different properties, a wall containing conductive materials, and a wall with cavities, an accurate detection of heartbeat was not possible in experiments using these kinds of wall.

## 6. Conclusion

In this paper, a new method to extract the human heart rate through a wall using UWB impulse radar is proposed. Unlike conventional methods that process the radar signal in the frequency domain for extracting the heartbeat information from the radar signal reflected from the human body, for this method, the radar signal was processed in the time domain. To accomplish this, a frame set was made by accumulating radar pulses at regular time intervals. The frame set was then converted to binary form, and the heartbeat information was extracted from the PCA of the time series data that indicated the presence of the heartbeats. Experiments were conducted to verify the performance of the proposed method for various subjects. In the experiments, the frequencies of the heart rates of subjects located within 1 m from the antenna were extracted, with an average error rate of 1.05%, and it was confirmed that instantaneous changes in the heartbeats could be detected with the proposed method.

In conclusion, the proposed method, which has a performance differentiated from other methods discussed above, can be used to extract heart rates through walls using radar signals.
